# Prevalence and associated factors of gestational diabetes mellitus among women with polycystic ovary syndrome: a systematic review and meta-analysis

**DOI:** 10.3389/fendo.2026.1874468

**Published:** 2026-07-17

**Authors:** Youchang Yuan, Liuxin Hu, Yanjing Li, Xinju Hou

**Affiliations:** 1 Department of Rehabilitation Medicine,Nanchang Hongdu Hospital of Traditional Chinese Medicine, Nanchang, Jiangxi, China; 2 Graduate School, Jiangxi University of Chinese Medicine, Nanchang, Jiangxi, China

**Keywords:** gestational diabetes mellitus, meta-analysis, polycystic ovary syndrome, related factors, systematic review

## Abstract

**Objective:**

Polycystic ovary syndrome (PCOS), the most common endocrine disorder in reproductive-aged women, often leads to gestational diabetes mellitus (GDM). This study aimed to systematically evaluate GDM prevalence and its associated factors in PCOS.

**Methods:**

We searched PubMed, Embase, Web of Science, Cochrane Library, CNKI, Wanfang, Sinomed, and VIP from inception to December 31, 2025. Studies reporting GDM prevalence or associated factors in PCOS were included. A random-effects model pooled prevalence with odds ratios. Egger’s test assessed publication bias, and subgroup analysis with meta-regression explored heterogeneity. A logit transformation was applied for pooling.

**Results:**

Twenty-two studies with 5,507 PCOS patients were included. The pooled GDM prevalence was 24% (95% CI: 20%-28%; prediction interval: 10%-46%), with substantial heterogeneity (I² = 89.9%). The primary prevalence estimate, based on cohort studies (n=13), was 21.7% (95% CI: 17.7%-25.8%), while the overall estimate including case-control studies was 24% (95% CI: 20%-28%). By diagnostic criteria, GDM prevalence was 26.0% for one-step vs. 18.7% for two-step. Potentially associated factors included pre-pregnancy BMI (OR = 1.39), gestational weight gain (OR = 1.58), HOMA-IR (OR = 3.41), and family history of diabetes (OR = 2.88).

**Conclusion:**

Although the pooled GDM prevalence in PCOS was 24%, substantial heterogeneity and a wide prediction interval (10%-46%) suggest the true prevalence varies considerably across settings. Associated factors included HOMA-IR, family history, pre-pregnancy overweight, and excessive gestational weight gain. PCOS patients should be considered a high-risk group, and early monitoring of glucose metabolism and weight management may be beneficial, though these recommendations derive from observed rather than interventional evidence.

**Systematic review registration:**

https://www.crd.york.ac.uk/PROSPERO/view/CRD42026128476, identifier: CRD420261284765.

## 1 Introduction

The prevalence of polycystic ovary syndrome (PCOS) among women globally is approximately 8% to 13% (1), positioning it as the most frequent endocrine and metabolic disorder in women of reproductive age. In addition to reproductive concerns such as ovulatory failure and hyperandrogenism, PCOS involves numerous metabolic disorders, the most prominent of which are insulin resistance (IR) and compensatory hyperinsulinemia. These underlying metabolic disorders often worsen during pregnancy, significantly increasing the risk of negative consequences for both mother and infant (2). Gestational diabetes mellitus (GDM) is recognized as one of the most frequently encountered pregnancy-associated disorders in women with PCOS (3). GDM leads to fetal macrosomia, neonatal hypoglycemia, and an increased risk of metabolic syndrome in offspring (4). Pregnant women are also at a higher risk of developing preeclampsia, cesarean delivery, type 2 diabetes mellitus, and cardiovascular disease over time. GDM is one of the most common pregnancy complications among PCOS patients. To develop targeted screening tools and management strategies, it is crucial to accurately estimate GDM prevalence and its associated factors among PCOS patients.

In recent years, various observational studies have examined the risk of gestational diabetes in PCOS patients, as well as potential contributing factors such as advanced maternal age, elevated body mass index (BMI), use of assisted reproductive technology, and hyperandrogenism. However, there is significant diversity in the outcomes due to differences in study design, demographic characteristics, and diagnostic criteria. Previously published meta-analyses that have preliminarily examined the association between PCOS and GDM have significant limitations (5, 6), including a small number of included studies; a lack of systematic comparisons across subgroups—particularly with regard to diagnostic criteria; and an inadequate investigation into the sources of heterogeneity.

Thus, the goal of this study was to quantitatively synthesize the key factors influencing this complication, thoroughly evaluate the pooled prevalence of GDM among PCOS patients through a systematic review and meta-analysis, and investigate potential sources of heterogeneity through subgroup analysis and meta-regression. The aims are to identify high-risk groups, provide more detailed epidemiological data for clinical practice, and provide evidence-based medicine support for the development of early intervention methods and GDM screening criteria.

## 2 Methods

### 2.1 Examine registration and reporting standards

The protocol for this systematic review was prospectively registered in the PROSPERO database (registration number: CRD420261284765) before data extraction and analysis, in accordance with the Preferred Reporting Items for Systematic Reviews and Meta-Analyses (PRISMA) 2020 guidelines (7).

### 2.2 Literature search strategy

From the beginning to December 31, 2025, we examined eight electronic data sources, including PubMed, Web of Science, Embase, and Cochrane Library for international studies, as well as SinoMed, CNKI, Wanfang Database, and VIP for Chinese studies, limiting the study type to human research and language to either Chinese or English. We employed a search strategy that integrated Medical subject headings (MeSH) with free text terms. The keywords used were “polycystic ovary syndrome,” “PCOS,” “gestational diabetes mellitus,” “GDM,” “prevalence,” “morbidity,” “risk factors,” and “influencing factors.” **Supplementary Table 1** provides detailed search strategies for all databases.

### 2.3 Inclusion and exclusion criteria

Inclusion criteria: (1) patients with a confirmed diagnosis of polycystic ovary syndrome and gestational diabetes, based on explicit diagnostic criteria such as ICD codes or recognized clinical diagnostic standards; (2) study into gestational diabetes prevalence and/or risk factors in PCOS patients; (3) only cross-sectional, cohort, or case-control studies are permitted.

Exclusion criteria: (1) research where data are duplicated or cannot be extracted; (2) case reports, reviews, animal experiments, and conference papers; (3) Studies not published in Chinese or English; (4) Studies lacking accessible full texts.

For clarity of diagnostic classification, the diagnostic criteria for PCOS and GDM applied in the included studies were explicitly defined as follows: PCOS diagnosis was based on either the Rotterdam criteria (2003) or the Chinese criteria (including the Chinese PCOS diagnostic guidelines, the Chinese PCOS diagnostic industry standard, or the endocrinology expert consensus on diagnosis and management of PCOS). GDM diagnosis was categorized into one-step and two-step methods. The one-step method referred to a single 75-g (or 100-g) oral glucose tolerance test (OGTT) using the IADPSG 2010 criteria or the Chinese Guidelines 2014 criteria (fasting ≥5.1 mmol/L, 1h ≥10.0 mmol/L, 2h ≥8.5 mmol/L), with GDM diagnosed if any single time point exceeded the threshold. The two-step method involved an initial 50-g glucose challenge test (GCT) screening (1h glucose ≥7.2–7.8 mmol/L), followed by a 100-g OGTT if the screening result was positive, with diagnosis based on the Carpenter-Coustan or NDDG criteria (requiring two or more elevated values).

### 2.4 Literature screening and data collection

Following the deletion of duplicate studies, two researchers conducted a preliminary screening, reviewing abstracts and titles and removing papers that clearly did not meet the inclusion criteria. For studies considered potentially eligible after the preliminary screening, full texts were retrieved for careful reading and secondary screening. Data extraction used standardized tables, and the primary extracted data includes the study title, first author, year of publication, nation, study design, sample size, diagnostic criteria, prevalence statistics, effect sizes for various influencing factors, and their adjustment factors. The extraction process was performed individually by two investigators, after which cross-validation of the data was conducted to ensure consistency and correctness. To avoid overlapping populations, we reviewed study affiliations, periods, and inclusion criteria; when multiple studies appeared to share the same cohort, only the one with the largest sample size was retained. The two judges’ disputes were resolved through discussion during the literature screening and data extraction procedure. If there was no consensus, a third reviewer was called upon to make the ultimate decision.

### 2.5 Quality and bias assessment

Cross-sectional studies were assessed using the criteria established by the Agency for Healthcare Research and Quality (AHRQ) (8). The Newcastle-Ottawa Scale (NOS) (9) was used to assess cohort and case control studies. Two researchers did the quality assessment method independently. Each evaluation item was thoroughly reviewed and scored. Disagreements were settled through discussion. To ensure the objectivity and credibility of the quality rating results, a third researcher was engaged if disputes persisted after discussions.

### 2.6 Statistical analysis

The prevalence was calculated as the raw proportion of the number of people with PCOS and GDM divided by the total number of people diagnosed with PCOS. A logit transformation was used to stabilize variances and normalize the distribution of prevalence estimates before pooling, and the pooled prevalence with its 95% confidence interval was back-transformed to the original scale for reporting. Given that case-control studies are not ideal for prevalence estimation, we performed subgroup analysis by study design to compare estimates across different study types. The cohort-based estimate was considered more reflective of population-level prevalence. For related factors, a combined assessment was performed using the odds ratio (OR) and its 95% confidence interval (CI), and meta-analyses of all influencing factors included only studies that reported the adjusted OR after multivariate adjustment. It should be noted that the adjustment variables differed across the included studies (**Supplementary Table 5**). Therefore, the pooled ORs represent summary association estimates rather than independent effects, and the term “independent risk factors” is not used in this context. To analyze the sources of heterogeneity, we did subgroup analyses based on nation, study type, PCOS diagnostic criteria, and gestational diabetes diagnostic criteria. We sought to better understand the drivers of heterogeneity. To this end, additional meta-regression analyses were carried out. Sensitivity analysis was performed using the elimination procedure, and publication bias was assessed using the funnel plot and Egger’s test to ensure the robustness and dependability of the meta-analysis results. All statistical analyses were carried out using R 4.5.0.

## 3 Results

### 3.1 Study selection process and outcomes

**Figure 1:**
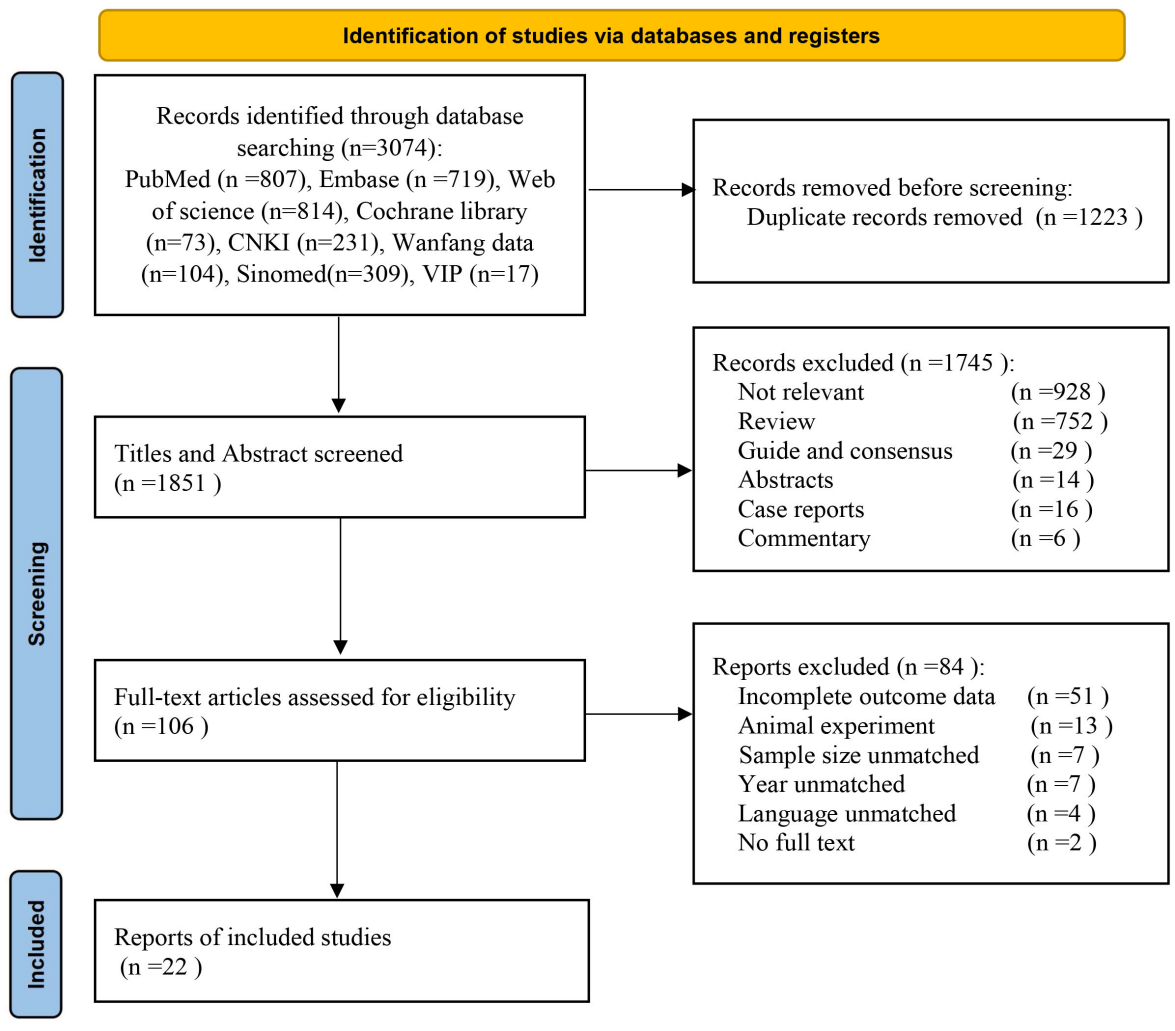
Flow diagram of the searching and screening.

When the initial search of 3074 articles was conducted, 1223 duplicate articles were excluded. Titles and abstract screening then led to the exclusion of an additional 1829 articles. After a full-text review of the remaining 106 papers, 22 papers were accepted and 84 were rejected (**Figure 1**).

### 3.2 Characteristics of the included studies

The 22 studies included 13 cohort studies, 7 case-control studies, and 2 cross-sectional studies. There were 17 studies from China, 3 from India, and 1 each from the United States and the Netherlands, respectively. Sample sizes across the included studies ranged from 35 to 988, comprising a total of 5507 participants. **Table 1** shows detailed information.

**Table 1:** Characteristics of studies included in this meta-analysis.

Author (year)	Subject	Case	Region	Study type	PCOS_criteria	GDM_criteria	NOS/AHRQ
Lu,YJ(2023) (30)	337	116	China	case-control study	Rotterdam criteria	One-step criteria	8
Sun,QX(2016) (31)	507	89	China	cohort study	Rotterdam criteria	One-step criteria	5
Huang,QF(2022) (32)	125	40	China	case-control study	Rotterdam criteria	One-step criteria	7
Hu,P(2015) (33)	73	11	China	cohort study	Rotterdam criteria	One-step criteria	7
Fan,ZN(2022) (34)	143	43	China	cohort study	Chinese criteria	One-step criteria	7
Jiang,XY(2025) (35)	106	37	China	case-control study	Chinese criteria	One-step criteria	7
Sun,CC(2020) (36)	114	54	China	case-control study	Chinese criteria	One-step criteria	7
Li,XZ(2018) (37)	35	12	China	case-control study	Rotterdam criteria	Two-step criteria	4
Liu,YJ(2025) (38)	174	52	China	cohort study	Chinese criteria	One-step criteria	6
Li,Y(2020) (39)	331	98	China	case-control study	Rotterdam criteria	One-step criteria	7
Zhang,N(2015) (40)	120	14	China	case-control study	Rotterdam criteria	Two-stepcriteria	5
Liu,XB(2024) (41)	104	31	China	cohort study	Chinese criteria	One-step criteria	6
Kumari,S.(2023) (42)	200	36	India	cohort study	Rotterdam criteria	One-step criteria	7
Li,G.(2018) (43)	248	75	China	cohort study	Rotterdam criteria	One-step criteria	6
Salgotra,M.(2024) (44)	300	56	India	cohort study	Rotterdam criteria	One-step criteria	6
Agnes Vijaya,U.(2015) (45)	56	8	India	cohort study	Rotterdam criteria	Two-stepcriteria	6
Zheng,W(2019) (46)	242	73	China	cohort study	Rotterdam criteria	One-step criteria	8
de Wilde,M.A.(2017) (13)	188	43	Holland	cohort study	Rotterdam criteria	Two-stepcriteria	9
Zhang,YJ(2016) (47)	268	45	China	cross-sectional study	Rotterdam criteria	One-step criteria	5
Xiao,Q(2016) (48)	652	64	China	cohort study	Rotterdam criteria	One-step criteria	7
Lo,J. C.(2017) (49)	988	192	America	cohort study	Rotterdam criteria	Two-stepcriteria	8
Li,X(2021) (50)	196	47	China	cross-sectional study	Rotterdam criteria	One-step criteria	5

One-step method: refers to directly performing a 75 g OGTT (or 100 g OGTT as a one-step diagnostic approach), using the IADPSG 2010 criteria or the Chinese Guidelines 2014 criteria (fasting ≥5.1, 1 h ≥10.0, 2 h ≥8.5 mmol/L), with a diagnosis made if any single time point exceeds the threshold; Two-step method: refers to first performing a 50 g GCT screening (1 h blood glucose ≥7.2–7.8 mmol/L), followed by a 100 g OGTT if the result is positive, with diagnosis based on the Carpenter-Coustan criteria or the NDDG criteria (typically requiring two or more values above the threshold); Chinese criteria include the Chinese guidelines for the diagnosis of polycystic ovary syndrome (PCOS), the Chinese industry standard for the diagnosis of PCOS, and the expert consensus on the endocrinological diagnosis and management of polycystic ovary syndrome.

### 3.3 Quality assessment results

The AHRQ checklist evaluates the quality of cross-sectional research, which consists of 11 elements responded to as “yes,” “no,” or “unclear,” with a total score range of 0-11. The overall scores of the studies were divided into three categories: Low, medium, and high quality are represented by ratings of at least three, four to seven, and eight to eleven, respectively. Cohort studies and case-control studies were assessed for quality using the NOS, which evaluates studies across three dimensions: selection of study populations, comparability, and assessment of exposure or outcome. A score of less than four indicates poor quality; five to six points indicate intermediate quality; and seven to nine points indicate good quality. Among the 22 studies incorporated into the analysis, 12 were rated as being of exceptional quality, 9 as moderate, and 1 as low quality. Detailed quality assessment scores for each study are presented in **Supplementary Table 2** (cross-sectional studies, AHRQ checklist), **Supplementary Table 3** (cohort studies, NOS), and **Supplementary Table 4** (case-control studies, NOS).

### 3.4 Prevalence of gestational diabetes in polycystic ovary syndrome

**Figure 2:**
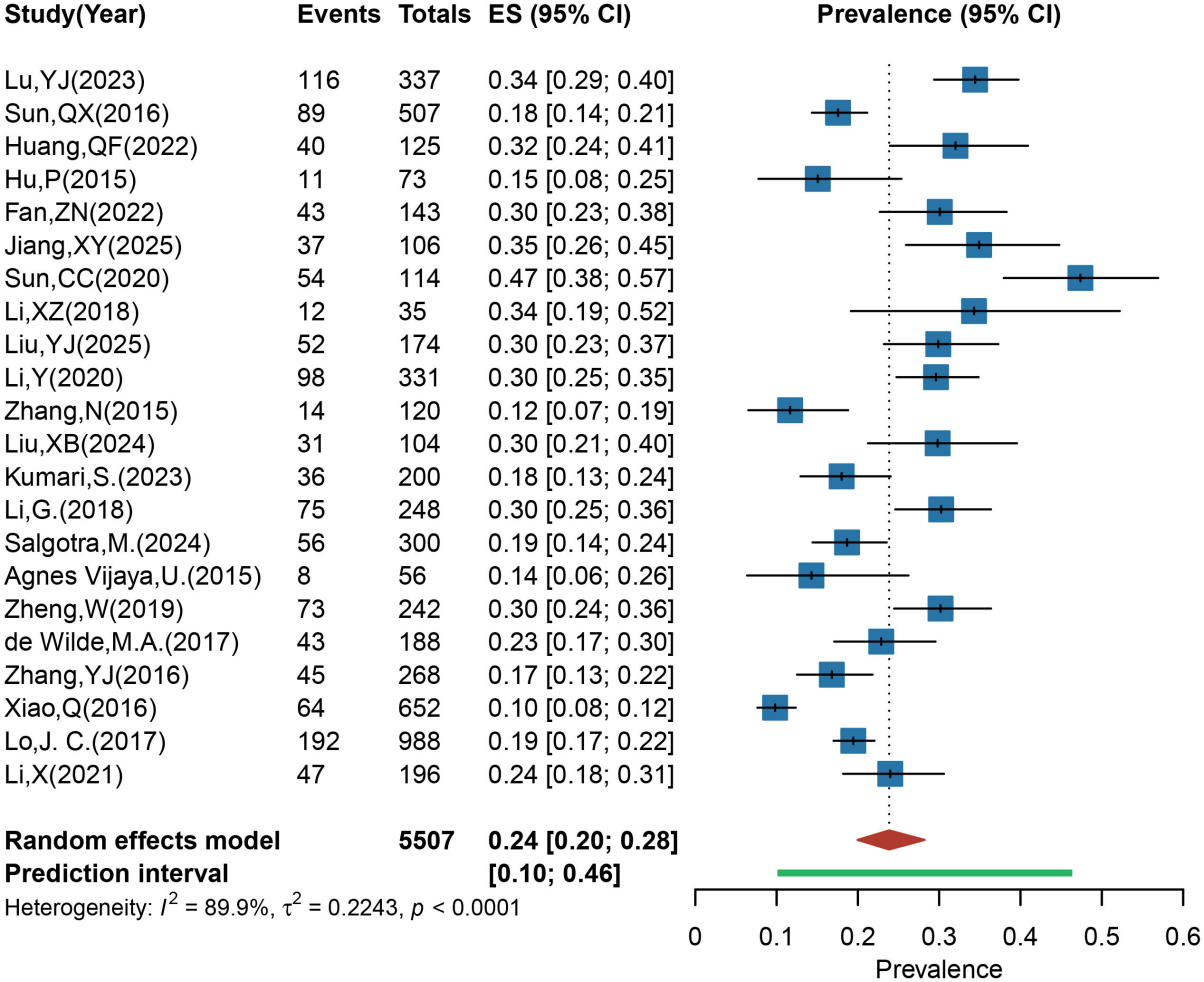
Forest plot from the meta-analysis of gestational diabetes prevalence. The overall pooled estimate (24%) includes all 22 studies (13 cohort, 7 case-control, and 2 cross-sectional studies). The cohort-based estimate (21.7%) is presented as the primary reference in the main text.

The included studies demonstrated significant heterogeneity (I² = 89.9%, P < 0.001). A random-effects model was utilized to conduct the pooled analyses. The pooled prevalence of GDM among PCOS patients was 24% (95% CI: 20%-28%) (**Figure 2**). However, substantial heterogeneity was present (I² = 89.9%, p < 0.001), indicating that the included studies did not estimate a single common underlying prevalence. The 95% prediction interval for the true prevalence in a future study ranged from approximately 10% to 46%, suggesting that the expected prevalence could vary dramatically across different populations or settings. Notably, cohort studies, which are less susceptible to selection bias than case-control studies, yielded a lower prevalence estimate of 21.7% (95% CI: 17.7%-25.8%), which may better approximate the true population-level GDM burden in PCOS patients.

#### 3.4.1 Subgroup analysis

We conducted pre-specified subgroup analyses stratified by country, study type, PCOS diagnostic criteria, and gestational diabetes diagnostic criteria to explore potential explanations for the substantial heterogeneity. (**Table 2**). Countries were divided into two groups: China and other countries (India, the Netherlands, and the United States). The diagnostic criteria for PCOS were classified using the Rotterdam criteria and Chinese criteria. The Chinese criteria included the Chinese PCOS diagnostic guidelines, the Chinese PCOS diagnostic industry standard, and the endocrine expert consensus on the diagnosis and treatment of PCOS. The diagnostic criteria for GDM are divided into two categories: one-step and two-step methods.

**Table 2:** Subgroup analyses.

Subgroup	N	Prevalence	95%CI	I2	P value	Analysis model
Region						
China group	17	26.50%	21.3%-31.6%	93.2%	<0.001	random-effects
other countries group	5	19.20%	17.4%-21.1%	0.0%,	0.587	random-effects
Study type						
case-control study	7	31.60%	23.3%-40.0%	89.5%	<0.001	random-effects
cohort study	13	21.70%	17.7%-25.8%	89.8%	<0.001	random-effects
PCOS_Criteria						
Rotterdam criteria	17	21.90%	18.1%-25.7%	90.7%	<0.001	random-effects
Chinese criteria	5	34.1%%	27.9%-40.3%	65.1%	0.022	random-effects
GDM_Criteria						
One-step criteria	17	26.00%	21.2%-30.8%	92.9%	<0.001	random-effects
Two-stepcriteria	5	18.70%	13.8%-23.7%	68.3%	0.013	random-effects

**Figure 3:**
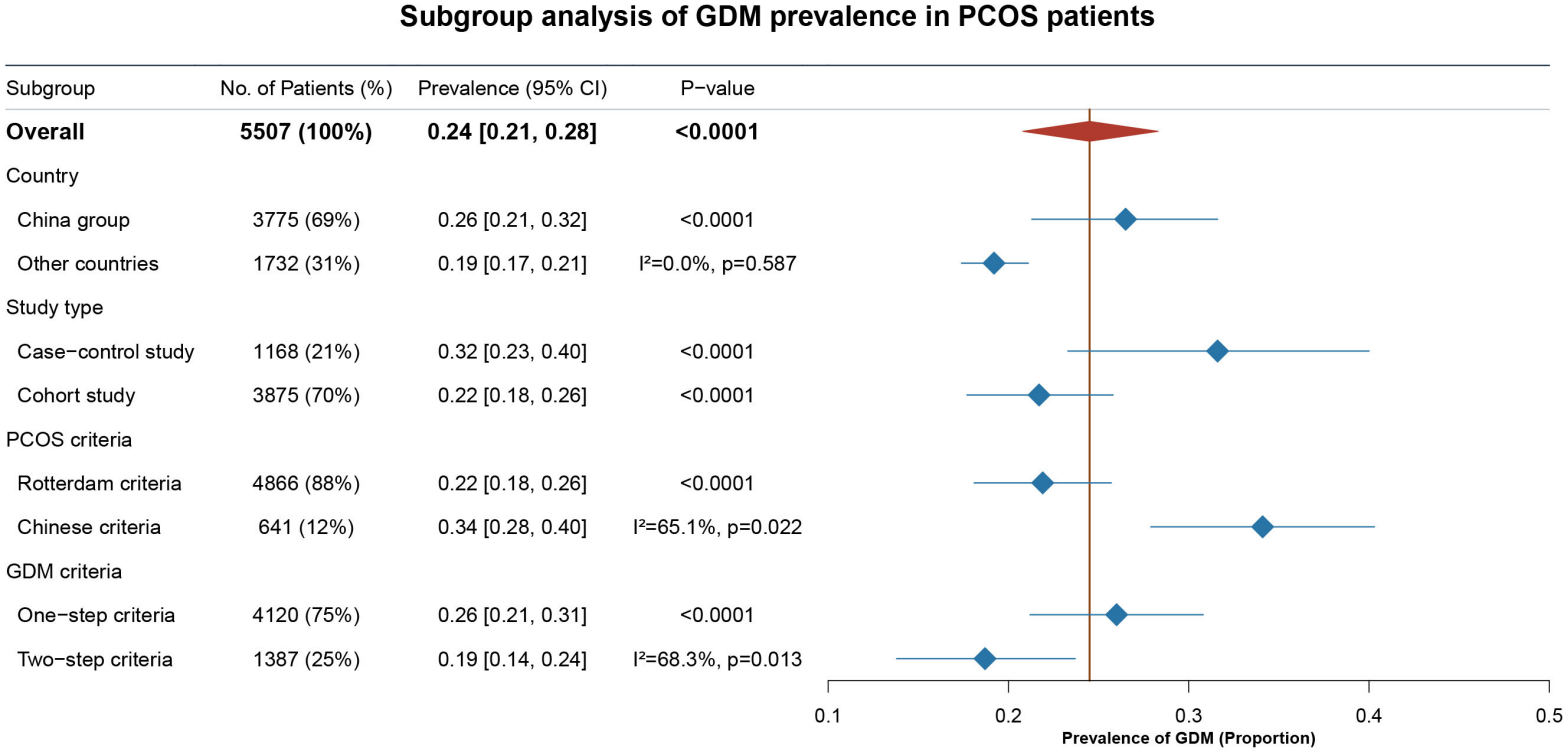
Subgroup analysis of gestational diabetes mellitus among polycystic ovary syndrome. Cross-sectional studies were not included in the subgroup analysis due to the limited number.

Among the 17 studies from China, the prevalence of GDM was 26.5% (95% CI: 21.3%-31.6%), with significant heterogeneity remaining (I²=93.2%, p<0.001). The pooled prevalence from the 5 studies conducted in other countries was 19.2% (95% CI: 17.4%-21.1%), with no heterogeneity detected across studies (I²=0.0%, p=0.587). By study design, the prevalence of GDM in case-control studies was 31.6% (95% CI: 23.3%–40.0%), significantly higher than the 21.7% (95% CI: 17.7%–25.8%) observed in cohort studies. Heterogeneity was high in both subgroups (case-control studies: I²=89.5%, p<0.001; cohort studies: I²=89.8%, p<0.001). Of note, only two cross-sectional studies were available, reporting prevalence rates of 16.8% and 23.98%, respectively. Given the limited number, these studies were included in the overall pooled estimate (**Figure 2**) but were not separately pooled as a subgroup. Regarding the diagnostic criteria for PCOS, the GDM prevalence was 21.9% (95% CI: 18.1%–25.7%) among patients diagnosed using the Rotterdam criteria, whereas the prevalence was significantly higher at 34.1% (95% CI: 27.9%–40.3%) among those diagnosed using the Chinese criteria. Heterogeneity within both subgroups decreased but remained substantial (Rotterdam criteria: I²=90.7%, p<0.001; Chinese criteria: I²=65.1%, p=0.022). This substantial difference (34.1% vs. 21.9%) likely reflects that the Chinese criteria identify a PCOS subgroup with more pronounced metabolic characteristics, including higher rates of insulin resistance and obesity, compared with the Rotterdam criteria. Consequently, the GDM risk associated with PCOS may vary considerably depending on which diagnostic criteria are used and which phenotypic subgroups are captured. Regarding the diagnostic criteria for GDM, the prevalence of GDM was 26.0% (95% CI: 21.2%–30.8%) for one-step diagnosis, which was higher than that for two-step diagnosis (18.7%, 95% CI: 13.8%–23.7%). The heterogeneity within the two subgroups was I²=92.9% (p<0.001) for the one-step method and I²=68.3% (p=0.013) for the two-step method, respectively (**Figure 3**).

#### 3.4.2 Meta-regression analysis

To explore possible sources of heterogeneity between studies, we performed meta-regression analyses. Regarding study design, case-control studies showed a significantly higher pooled effect size compared with cohort studies (β = 0.1108, 95% CI: 0.0168–0.2048, P = 0.021). Publication year was significantly and positively correlated with effect size (β = 0.0179, 95% CI: 0.0067–0.0292, P = 0.002), suggesting an increasing trend in the prevalence of GDM over time. However, this finding should be interpreted with caution given the limited number of studies. In contrast to the numerical differences observed in the subgroup analyses, the univariable analysis found no statistical significance (P > 0.05) for country, PCOS diagnostic criteria, GDM diagnostic criteria, or mean age. This could be attributed to sample size and confounding among factors. (**Table 3**).

**Table 3:** Meta-regression of gestational diabetes mellitus among polycystic ovary syndrome.

Variables	Coefficient	SE	P value	CI-lower	CI-upper
Region	0.0859	0.0531	0.1054	-0.0181	0.1899
Study Type	0.1108	0.048	0.0209	0.0168	0.2048
PCOS_Criteria	0.0859	0.0531	0.1054	-0.0181	0.1899
GDM_Criteria	0.0724	0.0557	0.1939	-0.0368	0.1815
Publication year	0.0179	0.0057	0.0017	0.0067	0.0292
Average age	0.0275	0.018	0.1268	-0.0078	0.0629

#### 3.4.3 Sensitivity analysis and publication bias

**Figure 4:**
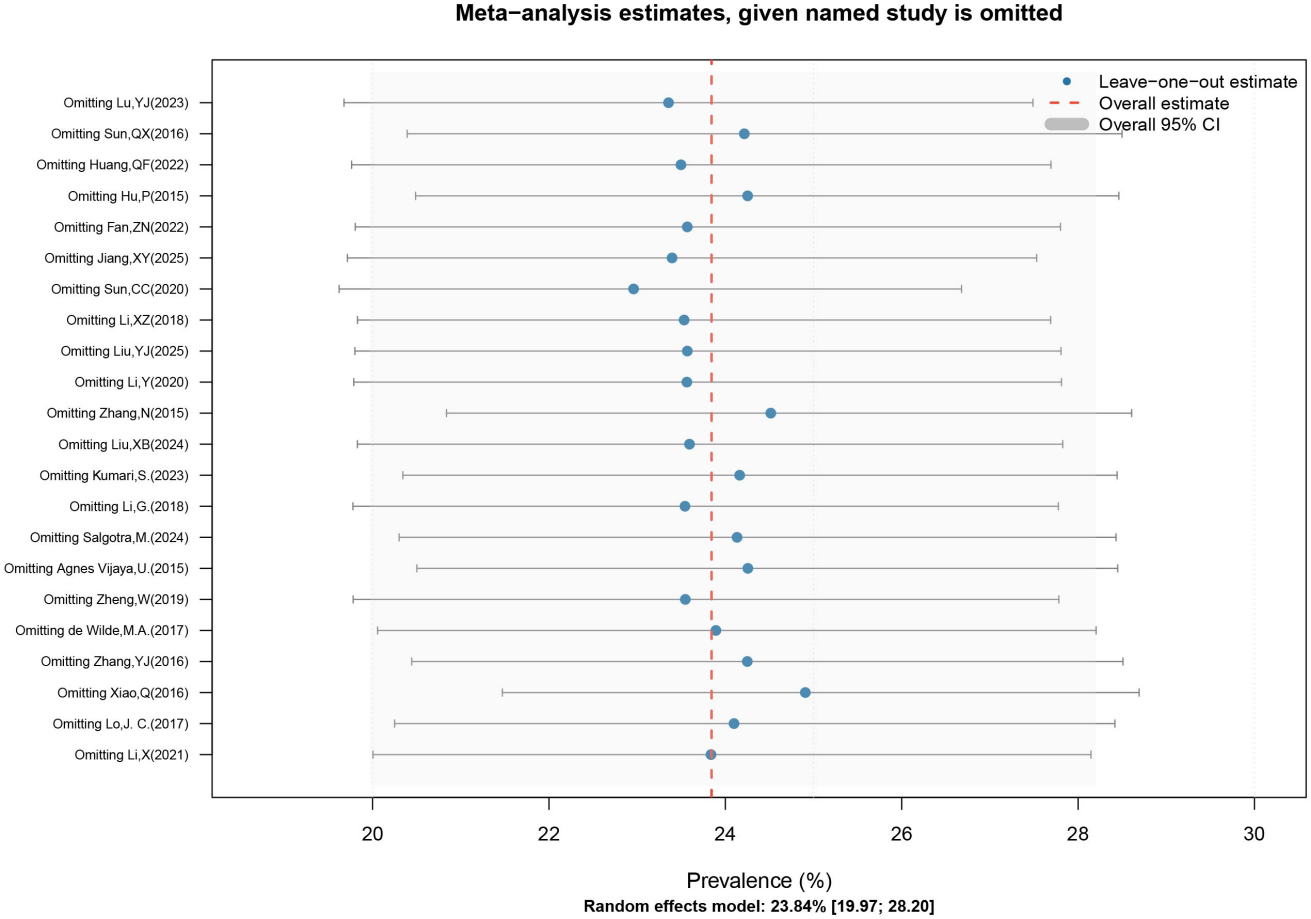
Sensitivity analysis for prevalence of gestational diabetes mellitus among polycystic ovary syndrome.

The Egger’s test and a funnel plot were employed to assess publication bias. There was no clear evidence of publication bias because the funnel plot showed a symmetric range of impact sizes surrounding the pooled estimate (**Supplementary Figure 1**). According to Egger’s test, the selected papers showed no statistically significant publication bias (P = 0.63). Nevertheless, Egger’s test is of limited appropriateness for proportion meta-analyses given the high heterogeneity (I² = 89.9%). For subgroup and risk-factor analyses with limited numbers of studies (3–4 studies per factor), publication bias assessment is underpowered and was therefore not performed. Sensitivity analysis revealed that deleting any one study had no meaningful influence on the total estimate (**Figure 4**).

### 3.5 Influencing factors

**Figure 5:**
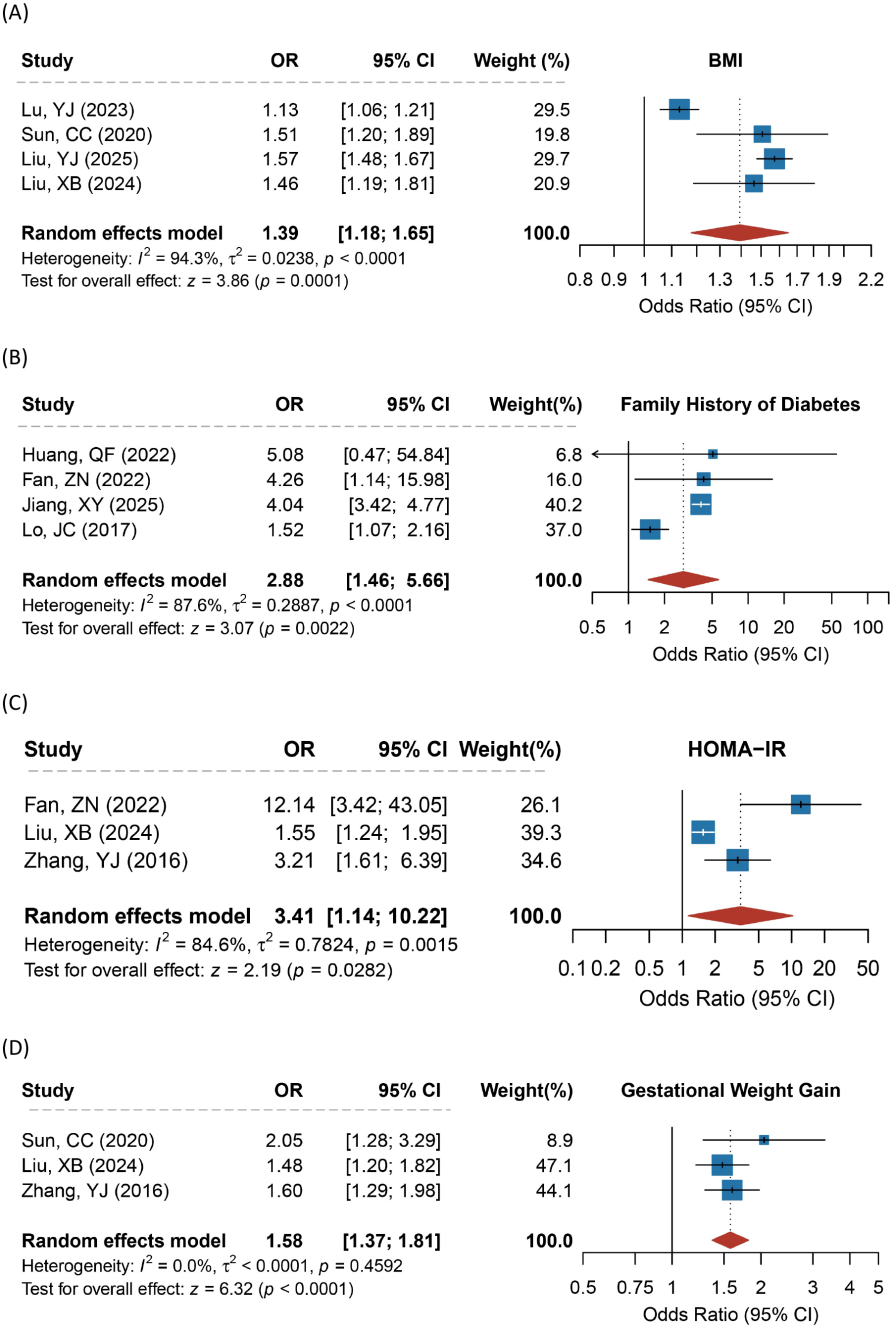
Pooled risk factors of gestational diabetes mellitus among polycystic ovary syndrome. **(A)** Body Mass Index **(B)** family history of diabetes **(C)** Homeostatic Model Assessment of Insulin Resistance **(D)** Gestational Weight Gain.

Insulin resistance is a key feature of PCOS. Three studies specifically examined how homeostatic model assessment of insulin resistance (HOMA-IR), a validated index of insulin resistance, relates to the subsequent risk of GDM in this patient population. The overall odds ratio (OR), according to the data, was 3.41 (95% CI: 1.14-10.22, P = 0.028), showing a considerably higher risk. Four studies were used to investigate the relationship between GDM prevalence in PCOS individuals and a family history of diabetes mellitus. To account for high heterogeneity (I² = 87.6%, P < 0.001), we employed a random-effects model in the meta-analysis. According to the research results, compared with patients without a family history, PCOS patients with a family history have a significantly higher risk of developing GDM, and there is a significant association between family history and diabetes history (OR = 2.88, 95% CI: 1.46–5.66). Regarding body composition-related indicators, data on preconception body mass index (BMI) (OR = 1.39, 95% CI: 1.18–1.65, P < 0.001) and gestational weight gain (OR = 1.58, 95% CI: 1.37–1.81, P < 0.001) were pooled using a random-effects model in 4 and 3 studies, respectively. The results showed that both indicators were significantly and positively associated with the risk of GDM in PCOS patients (**Figure 5**). However, given the small number of studies and high heterogeneity (I² > 80% for most factors), these findings should be interpreted as exploratory and require validation in future large-scale studies. Exposure definitions also varied across studies (e.g., BMI as continuous vs. categorical; different thresholds for gestational weight gain), limiting comparability.

## 4 Discussion

This systematic review and meta-analysis was performed to comprehensively estimate the population-based prevalence of GDM and its determinants in women with PCOS. This meta-analysis comprised 22 trials with 5,507 PCOS patients. The study’s findings indicate that PCOS may be a high-risk factor for GDM because the comorbidity rate of GDM in PCOS patients is 24% (95% CI: 20%-28%), which is higher than the upper limit of GDM prevalence in normal pregnant women (10%-25%) (4, 10) in previous research. Furthermore, this study found that HOMA-IR, gestational weight gain, pre-pregnancy BMI, and a family history of diabetes all contribute significantly to the development of GDM in PCOS. These findings reinforce the clinical recognition of PCOS as a high-risk marker for GDM and provide a quantitative basis for targeted screening and intervention.

The pooled GDM prevalence in our study (24%) is consistent with Dube et al. (6) (23.2%). Our analysis (22 studies, 5,507 patients) represents a larger sample size than prior meta-analyses and systematically quantified four risk factors (HOMA-IR, family history of diabetes, pre-pregnancy BMI, and gestational weight gain). Subgroup analyses showed that diagnostic criteria significantly influence prevalence estimates. Meta-regression suggested a positive association between publication year and GDM prevalence, though this finding should be interpreted with caution.

The subgroup analysis of this study showed that the prevalence of GDM among women with PCOS in China (26.5%) was higher than that in other countries (19.2%). The prevalence of β-cell dysfunction and insulin resistance is higher in the Chinese population, which may explain this difference, as even with a lower BMI (11), the risk of metabolic abnormalities is significantly higher. In recent years, the rapid increase in overweight and obesity rates in China has also explained this difference to some extent (12). Consistently, according to Chinese diagnostic criteria, the prevalence of GDM among PCOS patients (34.1%) is significantly higher than that in patients diagnosed with PCOS using the Rotterdam criteria (21.9%). Possible explanations include the following: The definition of clinical phenotypes in Chinese diagnostic criteria differs from the Rotterdam criteria, which may select a subgroup with more pronounced metabolic characteristics; notably, the hyperandrogenic phenotypes (classic PCOS) are associated with higher metabolic risk and greater insulin resistance compared with non-hyperandrogenic phenotypes (13). Furthermore, since all studies adopting Chinese criteria were conducted in mainland China, it is impossible to exclude confounding effects from population genetic susceptibility, environmental factors, or region-specific diagnostic practices. Compared with the two-step diagnostic method (18.7%), the one-step method has a significantly higher prevalence (26.0%) for GDM. The one-step method employs a single 75-gram OGTT with a lower diagnostic threshold, thereby improving the detection rate (14, 15). This finding is consistent with previous studies, indicating that according to the one-step diagnostic criteria, the prevalence of GDM is 1.03 to 3.78 (16) times higher than that of the two-step criteria. In case-control studies, the prevalence of GDM (31.6%) was significantly higher than that in cohort studies (21.7%). This difference reflects the impact of study design on prevalence estimates: case-control studies primarily adopt a retrospective design, which is susceptible to selection bias and recall bias, especially when data are collected retrospectively; whereas cohort studies employ a prospective design, thus providing a more accurate reflection of the incidence of GDM in a real-world setting (17). Therefore, the cohort-based estimate of 21.7% likely represents a more reliable reference for population-level prevalence. Meta-regression analysis revealed a positive association between publication year and GDM prevalence (β = 0.0179, P = 0.002), although this finding should be interpreted with caution as it may reflect changes in diagnostic criteria, population BMI, screening practices, or study characteristics rather than a true temporal increase. This trend may be attributed to various factors, including obesity and insulin resistance (18), increased awareness of GDM screening, and the widespread use of assisted reproductive technology (ART). ART has improved pregnancy rates in women with PCOS, but it may also increase the risk of GDM (19).

Regarding influencing factors, this study found that family history of diabetes (OR = 2.88) and HOMA-IR (OR = 3.41) showed the strongest associations with GDM in the meta-analysis, which are highly consistent (20) with existing concepts regarding genetic susceptibility and core pathological mechanisms of PCOS. During pregnancy, insulin sensitivity significantly decreases, leading to increased insulin resistance, and patients who have previously exhibited insulin resistance and compensatory β-cell dysfunction are at significantly higher risk of developing GDM (21). Clinical and animal studies have shown that insulin sensitizers like metformin reduce the risk of GDM in PCOS individuals (22, 23). Meanwhile, preconception overweight, and excessive weight gain during pregnancy also exhibit a significant positive association, both of which are modifiable risk factors. Obesity increases adipose tissue inflammation, insulin resistance, and lipotoxicity (24), thereby significantly elevating the risk of GDM.

IR, obesity, and genetic susceptibility do not act independently in the development of GDM among women with PCOS; rather, they form a synergistic pathological network. First, a bidirectional vicious cycle exists between obesity and IR. In the obese state, adipose tissue oversecretes leptin, resistin, and pro-inflammatory cytokines (e.g., TNF-α, IL-6), which activate the NF-κB and JNK signaling pathways, interfering with insulin receptor substrate (IRS) phosphorylation and thereby exacerbating peripheral IR (25). In turn, hyperinsulinemia promotes lipid synthesis and inhibits lipolysis, further aggravating visceral fat accumulation (26). Second, genetic susceptibility provides the background for this vicious cycle. Genome-wide association studies have identified multiple PCOS susceptibility loci, such as DENND1A, THADA, and FSHR, which are involved not only in follicular development but also in pancreatic β-cell function regulation and body fat distribution (27). Specific genetic backgrounds may amplify the burden of IR on β-cells and reduce their compensatory reserve capacity (28). Finally, pregnancy itself is a physiological state of IR, with placental hormones such as human placental lactogen, TNF-α, and progesterone antagonizing insulin signaling. For PCOS patients who already have pre-existing IR and compromised β-cell reserve, the metabolic stress of pregnancy more readily exceeds the compensatory threshold, thereby triggering GDM (29). Thus, IR, obesity, and genetic susceptibility constitute a “metabolic triple hit” that synergistically drives the transition from PCOS to GDM.

The conclusions of this study carry significant clinical implications. Pregnant women with PCOS, high BMI, or a family history should be classified as high-risk groups. These findings suggest that early monitoring of glucose metabolism and weight management may be beneficial in this population, though direct interventional evidence is needed to confirm whether these strategies reduce complications. Future research should focus on evaluating the precise efficacy of preconception weight loss and insulin-sensitizing therapy such as metformin in preventing GDM in patients with PCOS who have a high insulin resistance index and are overweight, through prospective cohort studies.

## 5 Limitations

Although this study has the advantages of comprehensive retrieval, large sample size, and multidimensional exploration of heterogeneity, several important limitations must be acknowledged. Substantial heterogeneity was present in the primary meta-analysis (I² = 89.9%, p < 0.001). While a random-effects model was used to accommodate this heterogeneity statistically, this approach does not make clinically heterogeneous studies comparable nor eliminate the threat to validity posed by such high heterogeneity. The wide prediction interval (10%-46%) indicates that the true prevalence of GDM in a new similar study could vary dramatically, and therefore the pooled estimate of 24% should be interpreted with considerable caution rather than as a definitive prevalence rate. Subgroup analyses and meta-regression identified study design, publication year, and geographic region as contributing factors. However, only univariable meta-regression was feasible due to the limited number of studies (n = 22), and confounding among moderators could not be fully examined. Significant residual heterogeneity remained, likely due to unmeasured confounders such as gestational diet, specific PCOS phenotypes, use of ovulation-inducing medications, and differences in screening protocols. The lack of phenotype-stratified data in the original studies prevented us from determining whether the observed GDM risk is uniformly elevated across all PCOS phenotypes. Furthermore, the risk-factor meta-analysis was limited by the small number of included studies (3–4 studies per factor), variation in exposure definitions across studies (e.g., BMI as continuous vs. categorical; different thresholds for gestational weight gain), and substantial heterogeneity (I² = 84.6% to 94.3%), reducing the precision and generalizability of the effect estimates. Furthermore, some of the included studies employed retrospective designs, which are inherently subject to recall and recording bias, thereby limiting the absolute establishment of causality. Language restriction to Chinese/English may also have introduced bias. These findings should therefore be considered exploratory, and future large-scale prospective studies are needed to validate the observed associations.

## 6 Conclusion

In conclusion, this study provides a pooled GDM prevalence estimate of 24% (95% CI: 20%-28%) among PCOS patients, though this estimate varied substantially by diagnostic criteria (26.0% for one-step vs. 18.7% for two-step). HOMA-IR, family history of diabetes, and body weight were identified as potentially associated factors, but these findings require validation in larger studies. Given the substantial heterogeneity (I² = 89.9%) and wide prediction interval (10%-46%), the prevalence estimate should be interpreted with caution. Future prospective studies with standardized criteria are needed to obtain more precise estimates. The cohort-based estimate of 21.7% (95% CI: 17.7%-25.8%) may better reflect the true population-level prevalence among PCOS patients. Of note, these clinical implications derive from observed associations and require confirmation in interventional studies.

## Data Availability

The original contributions presented in the study are included in the article/**Supplementary Material**. Further inquiries can be directed to the corresponding author.
