# Supplementary material for: Prevalence and associated factors of gestational diabetes mellitus among women with polycystic ovary syndrome: a systematic review and meta-analysis
Source: Front Endocrinol (Lausanne). 2026 Jul 17;17:1874468. doi: 10.3389/fendo.2026.1874468 (PMC13423630; doi:10.3389/fendo.2026.1874468)
Supplement: Supplementary file 1 [file DataSheet1.docx]

***Supplementary File 1***

1. **Supplementary Tables**

**Supplementary Table 1: Database search**

**A combined subject and free word search was conducted using the following Chinese and English databases: PubMed, Web of Science, Embase, Cochrane Library, Sinomed, China National Knowledge Infrastructure (CNKI), VIP Database, and WanFang Database.**

**Supplementary Table 1.** Search strategies for each database.

Each database search strategy is presented separately below.

| **PubMed** **(from the inception to December 31, 2025), n=807** | |
| --- | --- |
| #1 | "Polycystic Ovary Syndrome"[MeSH Terms] OR ("Polycystic Ovary Syndrome"[Title/Abstract] OR "ovary syndrome polycystic"[Title/Abstract] OR "syndrome polycystic ovary"[Title/Abstract] OR "polycystic ovarian syndrome"[Title/Abstract] OR "ovarian syndrome polycystic"[Title/Abstract] OR "polycystic ovary syndrome 1"[Title/Abstract] OR "sclerocystic ovarian degeneration"[Title/Abstract] OR |
| #2 | "Pregnancy"[MeSH Terms] OR "Pregnancy"[Title/Abstract] OR "Pregnancies"[Title/Abstract] OR "Gestation"[Title/Abstract] |
| #3 | "Diabetes Mellitus"[MeSH Terms] OR "Diabetes"[Title/Abstract] OR "diabetes insipidus"[Title/Abstract] OR "diet diabetic"[Title/Abstract] OR "prediabetic state"[Title/Abstract] OR "scleredema adultorum"[Title/Abstract] OR "glycation end products advanced"[Title/Abstract] OR "glucose intolerance"[Title/Abstract] OR "Gastroparesis"[Title/Abstract] |
| #4 | "Prevalence"[MeSH Terms] OR "Prevalence"[Title/Abstract] OR "Prevalences"[Title/Abstract] OR "point prevalence"[Title/Abstract] OR "point prevalences"[Title/Abstract] OR "prevalence point"[Title/Abstract] OR "period prevalence"[Title/Abstract] OR "period prevalences"[Title/Abstract] OR "prevalence period"[Title/Abstract] |
| #5 | "Epidemiology"[MeSH Terms] OR "Epidemiology"[Title/Abstract] OR "social epidemiology"[Title/Abstract] OR "Epidemiologies"[All Fields] OR "Epidemiology"[MeSH Subheading] OR "Epidemiology"[All Fields] OR "Epidemiology"[MeSH Terms] OR "epidemiology s"[All Fields] AND "Social"[Title/Abstract] OR "epidemiology social"[Title/Abstract] OR "social epidemiologies"[Title/Abstract] |
| #6 | "Incidence"[MeSH Terms] OR "Incidence"[Title/Abstract] OR "Incidences"[Title/Abstract] OR "incidence proportion"[Title/Abstract] OR "incidence proportions"[Title/Abstract] OR "proportion incidence"[Title/Abstract] OR "cumulative incidence"[Title/Abstract] OR "cumulative incidences"[Title/Abstract] OR "incidence cumulative"[Title/Abstract] |
| #7 | "Morbidity"[MeSH Terms] OR "Morbidities"[Title/Abstract] OR "Morbidity"[Title/Abstract] |
| #8 | "Factor"[Title/Abstract] OR "Risk"[Title/Abstract] OR "Risks"[Title/Abstract] OR "influence"[Title/Abstract] |
| #9 | #4 OR #5 OR #6 OR #7 OR #8 |
| #10 | #1 AND #2 AND #3 AND #9 |

| **Embase (from the inception to December 31, 2025), n=719** | |
| --- | --- |
| #1 | ovary polycystic disease'/exp OR 'ovary polycystic disease' AND [humans]/lim AND [embase]/lim OR 'cystic ovary':ti,ab,kw OR 'micropolycystic ovary':ti,ab,kw OR 'multiple follicle cyst':ti,ab,kw OR 'ovary polycystic syndrome':ti,ab,kw OR 'ovary, micropolycystic':ti,ab,kw OR 'ovary, polycystic':ti,ab,kw OR 'polycystic ovarian disease':ti,ab,kw OR 'polycystic ovary':ti,ab,kw OR 'polycystic ovary disease':ti,ab,kw OR 'polycystic ovary syndrome':ti,ab,kw OR 'stein cohen leventhal syndrome':ti,ab,kw OR 'stein leventhal disease':ti,ab,kw OR 'stein leventhal syndrome':ti,ab,kw OR 'syndrome stein leventhal':ti,ab,kw OR 'ovary polycystic disease':ti,ab,kw |
| #2 | ('pregnancy'/exp OR 'pregnancy') AND 'article'/it AND [humans]/lim AND [embase]/lim OR 'child bearing':ti,ab,kw OR 'childbearing':ti,ab,kw OR 'gestation':ti,ab,kw OR 'gravidity':ti,ab,kw OR 'intrauterine pregnancy':ti,ab,kw OR 'labor presentation':ti,ab,kw OR 'labour presentation':ti,ab,kw OR 'pregnancy maintenance':ti,ab,kw OR 'pregnancy trimesters':ti,ab,kw OR 'pregnancy':ti,ab,kw |
| #3 | ('diabetes mellitus'/exp OR 'diabetes mellitus') AND 'article'/it AND [humans]/lim AND [embase]/lim OR 'diabetes':ti,ab,kw OR 'diabetic':ti,ab,kw OR 'diabets':ti,ab,kw OR 'unspecified diabetes mellitus':ti,ab,kw OR 'diabetes mellitus':ti,ab,kw |
| #4 | ('prevalence'/exp OR 'prevalence') AND 'article'/it AND [humans]/lim AND [embase]/lim OR 'prevalence study':ti,ab,kw OR 'prevalence':ti,ab,kw |
| #5 | ('epidemiology'/exp OR 'epidemiology') AND 'article'/it AND [humans]/lim AND [embase]/lim OR 'epidemiologic methods':ti,ab,kw OR 'epidemiologic research':ti,ab,kw OR 'epidemiologic studies':ti,ab,kw OR 'epidemiologic survey':ti,ab,kw OR 'epidemiological research':ti,ab,kw OR 'epidemiometry':ti,ab,kw OR 'epidemiology':ti,ab,kw |
| #6 | ('incidence'/exp OR 'incidence') AND 'article'/it AND [humans]/lim AND [embase]/lim OR 'incidence rate':ti,ab,kw OR 'rate, incidence':ti,ab,kw OR 'incidence':ti,ab,kw |
| #7 | ('morbidity'/exp OR 'morbidity') AND 'article'/it AND [humans]/lim AND [embase]/lim OR 'disease frequency':ti,ab,kw OR 'disease incidence':ti,ab,kw OR 'disorder incidence':ti,ab,kw OR 'morbidity pattern':ti,ab,kw OR 'morbidity rate':ti,ab,kw OR 'morbidity risk':ti,ab,kw OR 'rate, morbidity':ti,ab,kw OR 'morbidity':ti,ab,kw |
| #8 | ('risk factor'/exp OR 'risk factor') AND 'article'/it AND [humans]/lim AND [embase]/lim OR 'relative risk':ti,ab,kw OR 'risk factors':ti,ab,kw OR 'risk factor':ti,ab,kw |
| #9 | #4 OR #5 OR #6 OR #7 OR #8 |
| #10 | #1 AND #2 AND #3 AND #9 |

| **Web of Science Search (from the inception to December 31, 2025), n=814** | |
| --- | --- |
| #1 | ”Ovary Syndrome, Polycystic” OR “Syndrome, Polycystic Ovary” OR “Polycystic Ovarian Syndrome” OR “Ovarian Syndrome, Polycystic” OR “Polycystic Ovary Syndrome 1” OR “Sclerocystic Ovarian Degeneration” OR “Ovarian Degeneration, Sclerocystic” OR “Sclerocystic Ovary Syndrome” OR “Stein-Leventhal Syndrome” OR “Stein Leventhal Syndrome” OR “Syndrome, Stein-Leventhal” OR “Sclerocystic Ovaries” OR “Ovary, Sclerocystic” OR “Sclerocystic Ovary” OR “Polycystic Ovary Syndrome” (Topic) |
| #2 | “Pregnancies” OR “Gestation” OR “Pregnancy” (Topic) |
| #3 | Diabetes Mellitus OR "Diabetes" OR "diabetes insipidus" OR "diet diabetic" OR "prediabetic state" OR "scleredema adultorum" OR "glycation end products advanced" OR "glucose intolerance" OR "Gastroparesis" (Topic) |
| #4 | Prevalence OR "Prevalences" OR "point prevalence" OR "point prevalences" OR "prevalence point" OR "period prevalence" OR "period prevalences" OR "prevalence period" (Topic) |
| #5 | Epidemiology OR "social epidemiology" OR "Epidemiologies" OR "social epidemiologies" (Topic) |
| #6 | Incidence OR "Incidences" OR "incidence proportion" OR "incidence proportions" OR "proportion incidence" OR "cumulative incidence" OR "cumulative incidences" OR "incidence cumulative" (Topic) |
| #7 | Morbidity OR "Morbidities" (Topic) |
| #8 | Factor OR "Risk" OR "Risks" OR "influence" (Topic) |
| #9 | #4 OR #5 OR #6 OR #7 OR #8 |
| #10 | #9 AND #1 AND #2 AND #3 |

| **Cochrane Libraby (from the inception to December 31, 2025), n=73** | |
| --- | --- |
| #1 | MeSH descriptor: [Polycystic Ovary Syndrome] explode all trees OR (Stein-Leventhal Syndrome):ti,ab,kw OR (Stein Leventhal Syndrome):ti,ab,kw OR (Polycystic Ovary Syndrome 1):ti,ab,kw OR (Polycystic Ovarian Syndrome):ti,ab,kw OR (Ovary Syndrome, Polycystic):ti,ab,kw OR (Ovarian Degeneration, Sclerocystic):ti,ab,kw OR (Sclerocystic Ovary Syndrome):ti,ab,kw OR (Syndrome, Polycystic Ovary):ti,ab,kw OR (Syndrome, Stein-Leventhal):ti,ab,kw OR (Ovarian Syndrome, Polycystic):ti,ab,kw OR (Sclerocystic Ovarian Degeneration):ti,ab,kw OR (Sclerocystic Ovary):ti,ab,kw OR (Ovary, Sclerocystic):ti,ab,kw OR (Sclerocystic Ovaries) OR (Polycystic Ovary Syndrome):ti,ab,kw |
| #2 | MeSH descriptor: [Pregnancy] explode all trees OR (Pregnancies):ti,ab,kw OR (Gestation) OR (Pregnancy):ti,ab,kw |
| #3 | MeSH descriptor: [Diabetes Mellitus] explode all trees OR (Diabetes Mellitus):ti,ab,kw |
| #4 | MeSH descriptor: [Prevalence] explode all trees OR (Period Prevalence):ti,ab,kw OR (Prevalence, Period):ti,ab,kw OR (Period Prevalences):ti,ab,kw OR (Prevalences):ti,ab,kw OR (Point Prevalence):ti,ab,kw OR (Prevalence, Point):ti,ab,kw OR (Point Prevalences) OR (Prevalence):ti,ab,kw |
| #5 | MeSH descriptor: [Epidemiology] explode all trees OR (Social Epidemiology):ti,ab,kw OR (Social Epidemiologies):ti,ab,kw OR (Epidemiologies, Social):ti,ab,kw OR (Epidemiology, Social):ti,ab,kw OR (Epidemiology):ti,ab,kw |
| #6 | MeSH descriptor: [Incidence] explode all trees OR (Proportion, Incidence):ti,ab,kw OR (Attack Rate):ti,ab,kw OR (Incidence Proportion):ti,ab,kw OR (Rate, Attack):ti,ab,kw OR (Incidence, Cumulative):ti,ab,kw OR (Attack Rates):ti,ab,kw OR (Incidence Proportions):ti,ab,kw OR (Cumulative Incidences):ti,ab,kw OR (Cumulative Incidence):ti,ab,kw OR (Rate, Secondary Attack):ti,ab,kw OR (Secondary Attack Rate):ti,ab,kw OR (Secondary Attack Rates):ti,ab,kw OR (Attack Rate, Secondary):ti,ab,kw OR (Rate, Person-time):ti,ab,kw OR (Person-time Rates):ti,ab,kw OR (Incidence Rates):ti,ab,kw OR (Person time Rate):ti,ab,kw OR (Rate, Incidence):ti,ab,kw OR (Person-time Rate):ti,ab,kw OR (Incidence Rate):ti,ab,kw OR (Incidences):ti,ab,kw OR (Incidence):ti,ab,kw |
| #7 | MeSH descriptor: [Morbidity] explode all trees OR (Morbidity):ti,ab,kw OR (Morbidities):ti,ab,kw |
| #8 | MeSH descriptor: [Risk Factors] explode all trees OR (Risk Factor):ti,ab,kw OR (Factor, Risk):ti,ab,kw OR (Risk Factors):ti,ab,kw |
| #9 | #4 OR #5 OR #6 OR #7 OR #8 |
| #10 | #1 AND #2 AND #3 AND #9 |

| **Sinomed (from the inception to December 31, 2025), n=309** | |
| --- | --- |
| #1 | "多囊卵巢综合征"[不加权:扩展] |
| #2 | ("硬化囊状性卵巢"[常用字段:智能] OR "Stein-Leventhal综合征"[常用字段:智能] OR "Sclerocystic卵巢变性"[常用字段:智能] OR "Sclerocystic卵巢综合征"[常用字段:智能] OR "多囊卵巢综合征"[常用字段:智能]) |
| #3 | #1 OR #2 |
| #4 | "妊娠"[不加权:扩展] |
| #5 | "妊娠"[常用字段:智能] OR "怀孕"[常用字段:智能] |
| #6 | #4 OR #5 |
| #7 | "糖尿病"[常用字段:智能] OR "GDM"[常用字段:智能] |
| #8 | "影响因素"[常用字段:智能] OR "危险因素"[常用字段:智能] OR "相关因素"[常用字段:智能] OR "患病率"[常用字段:智能] OR "发生率"[常用字段:智能] OR "检出率"[常用字段:智能] OR "现状"[常用字段:智能] OR "流行"[常用字段:智能] OR "调查"[常用字段:智能] |
| #9 | #3 AND #6 AND #7 AND #8 |

| **CNKI, n=231** | (TKA='患病率' OR TKA='检出率' OR TKA='发生率' OR TKA='现状' OR TKA='流行' OR TKA='调查') AND (TKA='糖尿病' OR TKA='GDM') AND (TKA='硬化囊状性卵巢' OR TKA='Stein-Leventhal综合征' OR TKA='Sclerocystic卵巢变性' OR TKA='Sclerocystic卵巢综合征' OR TKA='多囊卵巢综合征') AND (TKA='妊娠' OR TKA='怀孕') (TKA='prevalence' OR TKA='detection rate' OR TKA='incidence' OR TKA='status' OR TKA='epidemic' OR TKA='survey') AND (TKA='diabetes' OR TKA='GDM') AND (TKA='sclerocystic ovary' OR TKA='Stein-Leventhal syndrome' OR TKA='sclerocystic ovarian degeneration' OR TKA='sclerocystic ovary syndrome' OR TKA='polycystic ovary syndrome') AND (TKA='pregnancy' OR TKA='gestation') |
| --- | --- |
| **Wanfang Database, n=104** | 关键词=(硬化囊状性卵巢 OR Stein-Leventhal综合征 OR Sclerocystic卵巢变性 OR Sclerocystic卵巢综合征 OR 多囊卵巢综合征) AND 关键词=(糖尿病 OR GDM) AND 关键词=(患病率 OR 检出率 OR 发生率 OR 现状 OR 流行 OR 调查) AND 关键词=(怀孕 OR 妊娠)  Keywords=(sclerocystic ovary OR Stein-Leventhal syndrome OR sclerocystic ovarian degeneration OR sclerocystic ovary syndrome OR polycystic ovary syndrome) AND Keywords=(diabetes OR GDM) AND Keywords=(prevalence OR detection rate OR incidence OR status OR epidemic OR survey) AND Keywords=(pregnancy OR gestation) |
| **VIP, n=17** | M=(硬化囊状性卵巢 OR Stein-Leventhal综合征 OR Sclerocystic卵巢变性 OR Sclerocystic卵巢综合征 OR 多囊卵巢综合征) AND M=(患病率 OR 检出率 OR 发生率 OR 现状 OR 流行 OR 调查) AND M=(糖尿病 OR GDM)  M=(sclerocystic ovary OR Stein-Leventhal syndrome OR sclerocystic ovarian degeneration OR sclerocystic ovary syndrome OR polycystic ovary syndrome) AND M=(prevalence OR detection rate OR incidence OR status OR epidemic OR survey) AND M=(diabetes OR GDM) |

Note: TKA = Title, Keyword, Abstract field, M = Title, Keyword, Abstract field.

**Supplementary Table 2. Quality assessment of cross-sectional study**

| Study Included | Define the source of information (survey, record review). | List inclusion and exclusion criteria for exposed and unexposed subjects (cases and controls) or refer to previous publications. | Indicate time period used for identifying patients. | Indicate whether or not subjects were consecutive if not population-based. | Indicate if evaluators of subjective components of study were masked to other aspects of the participants. | Describe any assessments undertaken for quality assurance purposes (e.g.,test/retest of primary outcome measurements). | Explain any patient exclusions from analysis. | Describe how confounding was assessed and/or controlled. | If applicable, explain how missing data were handled in the analysis. | Summarize patient response rates and completeness of data collection. | Clarify what follow-up, if any, was expected and the percentage of patients for which incomplete data or follow-up was obtained. | Total | Quality |
| --- | --- | --- | --- | --- | --- | --- | --- | --- | --- | --- | --- | --- | --- |
| Zhang,YJ（2016） | Yes | Yes | Yes | Unclear | No | No | Unclear | Yes | No | No | Yes | 5 | Moderate |
| Li,X（2021） | Yes | Yes | Yes | Unclear | No | Yes | Unclear | Yes | No | No | Unclear | 5 | Moderate |

**Supplementary Table 3. Quality assessment of cohort studies**

| Study Included | Selection  (4 stars) | Comparability  (2 stars) | Outcome  (3 stars) | Total  (9 stars) | Quality |
| --- | --- | --- | --- | --- | --- |
| Sun,QX（2016） | ★★ | ★ | ★★ | 5 | Moderate |
| Hu,P（2015） | ★★★ | ★★ | ★★ | 7 | High |
| Fan,ZN（2022） | ★★ | ★★ | ★★★ | 7 | High |
| Liu,YJ（2025） | ★ | ★★ | ★★★ | 6 | Moderate |
| Liu,XB（2024） | ★★ | ★★ | ★★ | 6 | Moderate |
| Kumari,S.（2023） | ★★★ | ★★ | ★★ | 7 | High |
| Li,G.（2018） | ★★ | ★★ | ★★ | 6 | Moderate |
| Salgotra,M.（2024） | ★★ | ★ | ★★★ | 6 | Moderate |
| Agnes Vijaya,U.（2015） | ★★ | ★ | ★★★ | 6 | Moderate |
| Zheng,W（2019） | ★★★★ | ★ | ★★★ | 8 | High |
| de Wilde,M.A.（2017） | ★★★★ | ★★ | ★★★ | 9 | High |
| Xiao,Q（2016） | ★★ | ★★ | ★★★ | 7 | High |
| Lo,J. C.（2017） | ★★★ | ★★ | ★★★ | 8 | High |

**Supplementary Table 4. Quality assessment of case-control studies**

| Study Included | Selection  (4 stars) | Comparability  (2 stars) | Exposure  (3 stars) | Total  (9 stars) | Quality |
| --- | --- | --- | --- | --- | --- |
| Lu,YJ（2023） | ★★★★ | ★★ | ★★ | 8 | High |
| Huang,QF（2022） | ★★★ | ★★ | ★★ | 7 | High |
| Jiang,XY（2025） | ★★★★ | ★ | ★★ | 7 | High |
| Sun,CC（2020） | ★★★★ | ★ | ★★ | 7 | High |
| Li,XZ（2018） | ★ | ★ | ★★ | 4 | Low |
| Li,Y（2020） | ★★★★ | ★ | ★★ | 7 | High |
| Zhang,N（2015） | ★★ | ★ | ★★ | 5 | Moderate |

**Supplementary Table 5. Summary of adjusted confounders for risk factors included in the meta-analysis**

| Study | Risk Factor | Effect Estimate (95% CI) | Type | Confounders Adjusted For |
| --- | --- | --- | --- | --- |
| Lu, YJ (2023) | Pre-pregnancy BMI | 1.13 (1.05-1.20) | OR | HbA1c, FPG |
| Sun, CC (2020) | Pre-pregnancy BMI | 1.51 (1.20-1.89) | OR | Pre-pregnancy fasting insulin, pre-pregnancy testosterone |
| Liu, YJ (2025) | Pre-pregnancy BMI | HR = 1.57 (1.48-1.67) | HR | FT₄, T |
| Liu, XB (2024) | Pre-pregnancy BMI | 1.46 (1.19-1.81) | OR | Gestational weight gain, HOMA-IR, FINS |
| Huang, QF (2022) | Family history of diabetes | 5.08 (0.47-54.84) | OR | Age at menarche, pre-pregnancy obesity, pre-pregnancy metformin use, gestational weight gain, sleep duration |
| Fan, ZN (2022) | Family history of diabetes | 4.26 (1.14-15.98) | OR | T, SHBG, HOMA-IR, CysC |
| Jiang, XY (2025) | Family history of diabetes | 4.06 (3.39-4.73) | OR | Age, pre-pregnancy BMI, testosterone, luteinizing hormone, pre-pregnancy metformin use, waist-to-hip ratio |
| Lo, JC (2017) | Family history of diabetes | 1.52 (1.07-2.16) | OR | Age, race/ethnicity, prepregnancy BMI, preconception metformin use, fertility treatment (drugs/IVF), multiple gestation |
| Liu, XB (2024) | HOMA-IR | 1.55 (1.24-1.96) | OR | Pre-pregnancy BMI, gestational weight gain, FINS |
| Fan, ZN (2022) | HOMA-IR | 12.14 (3.42-43.05) | OR | T, SHBG, family history of diabetes, CysC |
| Zhang, YJ (2016) | HOMA-IR | 3.21 (1.61-6.39) | OR | Pre-pregnancy WHR, pre-pregnancy SHBG, gestation weight gain before 24 weeks |
| Sun, CC (2020) | Gestational weight gain | 2.05 (1.28-3.29) | OR | Pre-pregnancy fasting insulin, pre-pregnancy testosterone |
| Liu, XB (2024) | Gestational weight gain | 1.48 (1.21-1.82) | OR | Pre-pregnancy BMI, HOMA-IR, FINS |
| Zhang, YJ (2016) | Gestational weight gain | 1.60 (1.29-1.98) | OR | Pre-pregnancy WHR, pre-pregnancy SHBG, pre-pregnancy HOMA-IR |

Note: HR from Liu, YJ (2025) was treated as an approximate equivalent of OR for meta-analysis, which is acceptable when the outcome incidence is low to moderate.

1. **Supplementary Figures**

**Supplementary Figures 1: Funnel plot of publication bias**

**
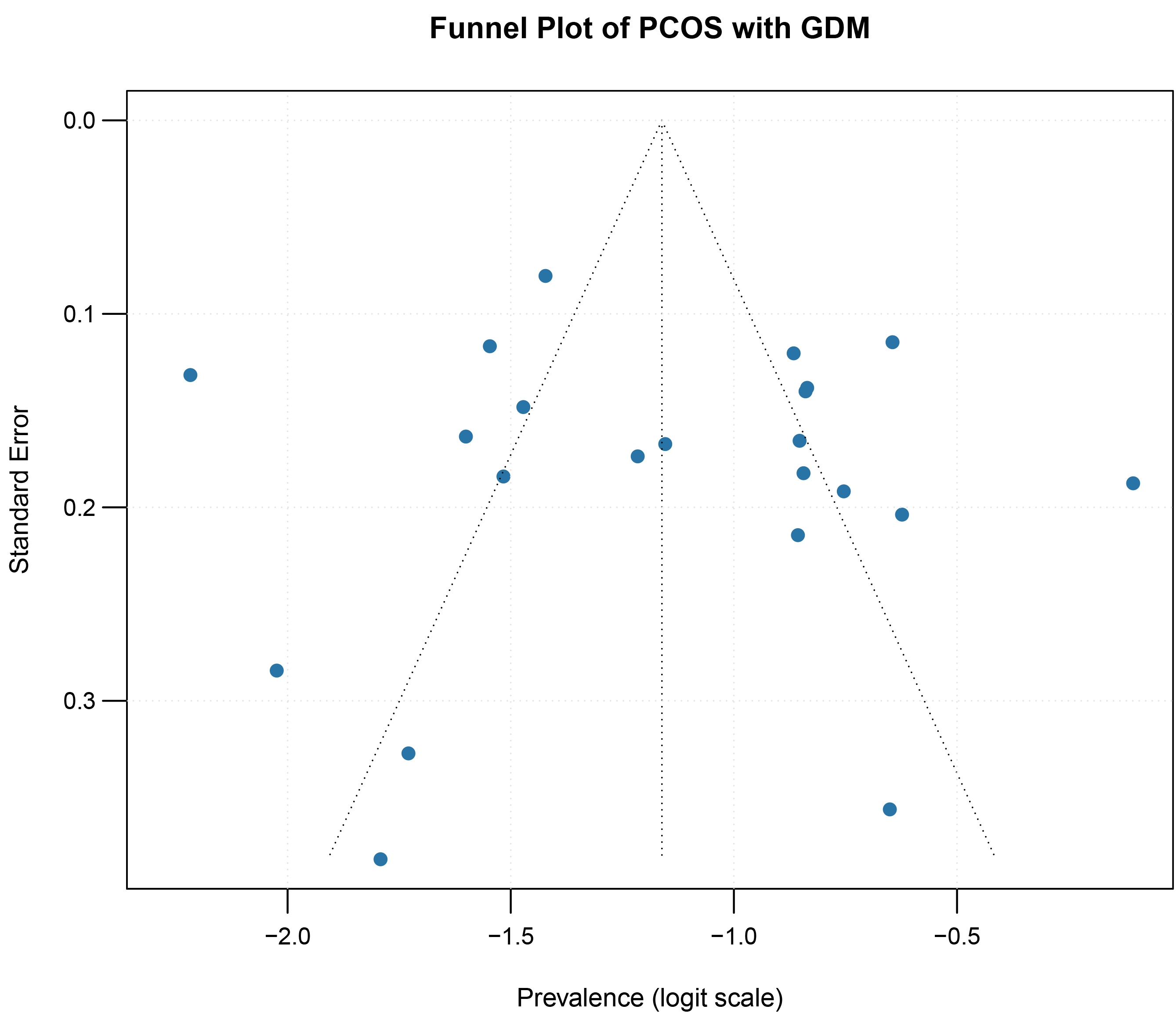
**
